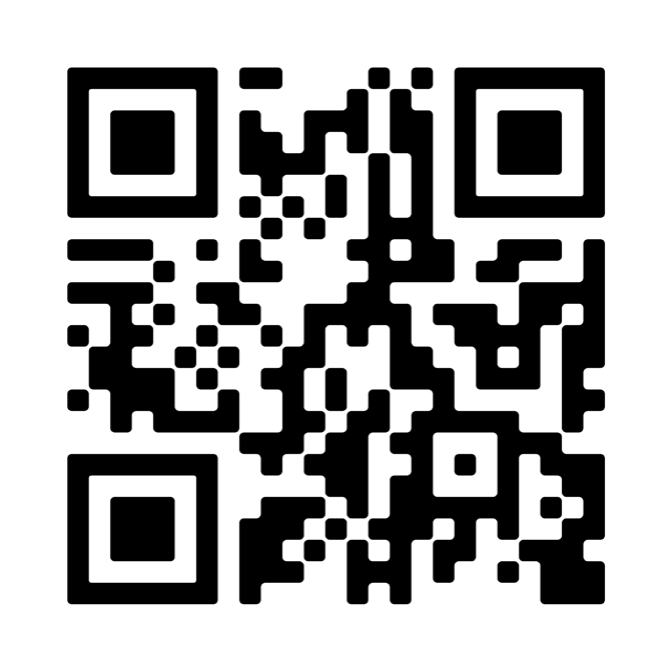# Laparoscopic management of a second trimester ruptured spontaneous heterotopic tubal pregnancy mimicking ovarian tumour, a case report

**DOI:** 10.52054/FVVO.15.2.069

**Published:** 2023-06-30

**Authors:** N Kathopoulis, M Diakosavvas, K Kypriotis, I Chatzipapas, E Domali, A Protopapas

**Affiliations:** 1^st^ Department of Obstetrics and Gynecology, University of Athens, Alexandra hospital, Athens, Greece

**Keywords:** Ectopic pregnancy, heterotopic pregnancy, laparoscopy

## Abstract

**Background:**

Laparoscopic surgery in the second trimester of pregnancy is a high risk and demanding operation. Especially when dealing with adnexal pathology, the surgeon should balance between the effort to establish adequate visualisation of the operating field with minimal uterine manipulation and use of energy application to avoid any potential adverse effects on the intrauterine pregnancy.

**Objective:**

The video shows laparoscopic surgery performed in the second trimester of pregnancy and highlights modifications to technique to ensure safety.

**Materials and Methods:**

We present a case report of spontaneous heterotopic tubal pregnancy that mimicked an ovarian tumour and was managed surgically with a laparoscopy in the second trimester. During surgery, a previously ruptured left tubal pregnancy (? ectopic) was the cause for a concealed hematoma in the pouch of Douglas, misdiagnosed as ovarian tumour. This is one of the few cases of heterotopic pregnancy treated by laparoscopy in the second trimester of pregnancy.

**Results:**

The patient was discharged the day 2 post-operatively, the intrauterine pregnancy progressed, and the patient delivered with a planned caesarean section on the 38th week.

**Conclusions:**

Laparoscopic surgery, with adjustments, is a safe and effective method to manage adnexal pathology during a second trimester pregnancy.

## Learning Objective

This video presents the laparoscopic management of a ruptured spontaneous heterotopic pregnancy at 17 weeks gestation, which mimicked a suspicious ovarian tumour. A step-by-step description of the modifications in the laparoscopic technique when operating in the second trimester is also discussed to help the viewer to reproduce this technique in similar demanding cases.

## Introduction

Heterotopic pregnancy (HP) is when an intrauterine and an ectopic pregnancy occur simultaneously. Recently, with the augmented application of assisted reproductive technique(ART), the rate has reportedly increased from 1/30000 up to 1/100 (Guan and Ma, 2017). Diagnosing HP is always a clinical challenge, as Serum beta-human Chorionic Gonadotropin levels (Bhcg) are of low diagnostic value due to the intrauterine pregnancy, and transvaginal sonography (TVS) is the mainstay of diagnosis. Unfortunately, early TVS is not always possible, and late diagnosis may be life-threatening and also impact viability of the intrauterine pregnancy. Surgical treatment is the first line treatment of HP as systemic methotrexate has the risk of possible teratogenic effects on the viable IUP. Recently, ultrasound-guided methotrexate use has been proven to be safe and effective to treat HP (Li et al., 2023). As laparoscopic surgical techniques improves, more challenging ectopic pregnancies can be managed this way (Kathopoulis et al., 2021). Laparoscopic salpingectomy or salpingotomy are appropriate to terminate the extrauterine pregnancy while reducing the risk posed to the intrauterine pregnancy (Torok et al., 2023).

In this case we describe a spontaneous heterotopic pregnancy diagnosed at 17 weeks gestation. There was concern regarding an ovarian tumour however on laparoscopy, this was revealed to be a tubal rupture which caused a concealed haematoma in the Douglas pouch. The patient recovered well post laparoscopy and obstetrically, the pregnancy continued up to term when a healthy girl was delivered by caesarean section. In this video we demonstrate easily reproducible steps and describe the techniques utilised, highlighting the critical points for the laparoscopic approach of adnexal pathology in the second trimester of pregnancy.

## Patients and methods

A 26-year-old woman, gravida 2 para 1, at 17 +2 days of gestation presented to Alexandra Hospital with a 3-day history of lower abdominal pain. She was afebrile, with no urinary symptoms or history of infectious contacts. She had no significant past medical history and was normally well. She did report experiencing similar pain at 9 weeks which resolved spontaneously, and she did not seek medical advice.

**Figure 1:**
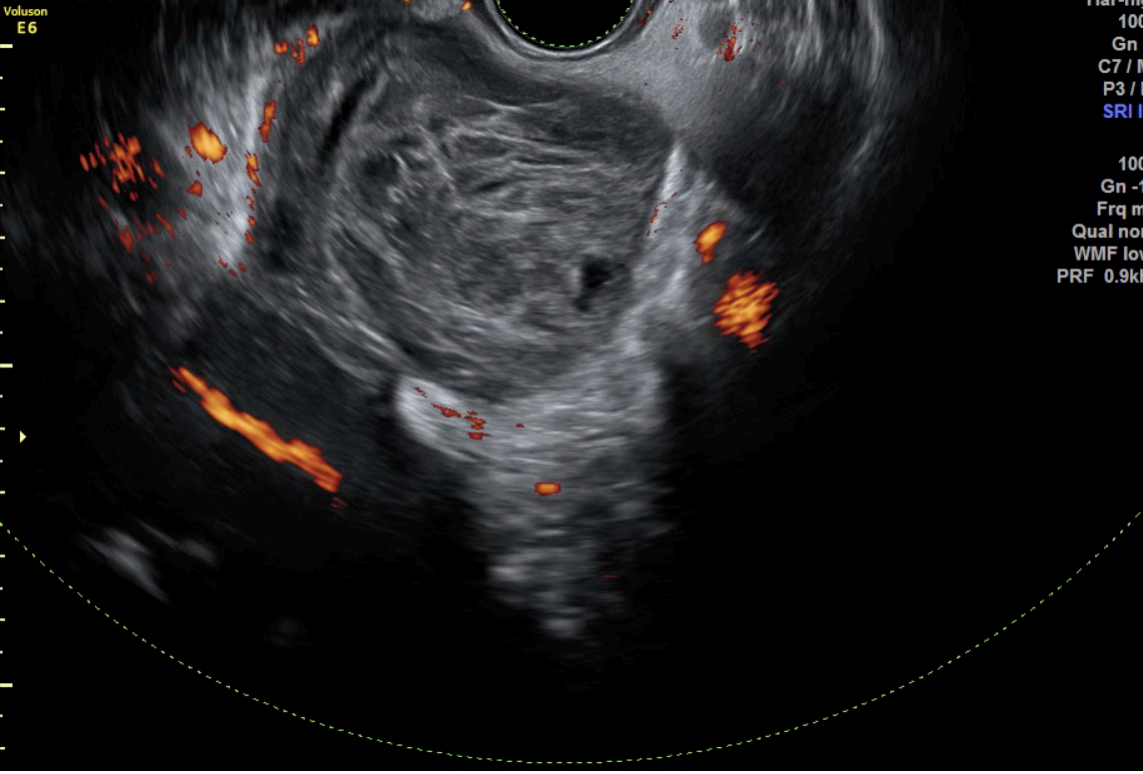
Vaginal ultrasound revealing a suspicious solid and cystic tumor possibly originating from the left ovary.

Transvaginal ultrasound showed a viable intrauterine pregnancy with biometer parameters within the normal range for that gestational age. It also revealed a 7 cm mass with solid and cystic components arising possibly from the left ovary and occupying the pouch of Douglas (Figure 1).

The mass was considered suspicious in appearance and therefore, tumour markers (CA-125 and CA 19-9) were performed which were within normal range. It was decided surgical management was most appropriate, especially in view of potential malignancy, and the laparoscopic approach was chosen.

## Results

The patient was positioned in dorsal lithotomy with pneumoperitoneum established using the Veress needle technique through Palmer’s point. The intrabdominal pressure was set at 20mmHg, and a 12mm umbilical trocar was introduced to insert the 10mm 30-degree angled laparoscope into the abdominal cavity. The upper abdominal organs were normal with the gravid uterus occupied 75% of the surgical field. The uterine fundus was just below the level of the umbilicus and an extra scoping trocar was inserted in the midline 4cm over the umbilicus. This allowed us to enlarge the view of the surgical field and facilitate the operation. Two accessory 5mm trocars were placed at the midclavicular line 5cm above the anterior superior iliac spine. Finally, a third 5mm trocar was placed at Palmer’s point allowing an extra instrument to access the left sided pathology. Subsequently, the intra-abdominal pressure was set at 12mmHg.

Using atraumatic forceps for blunt dissection and with limited use of bipolar energy, the surgeon initially attempted to identify the mass on the anatomical position of the left adnexa. Due to blood clots and plastron formation, there were solid adhesions with the sigmoid colon. With a careful technique, the adnexa was detached from the colon and the peritoneum of the left ovarian fossa. This revealed a normal ovary with a dilated fallopian tube, which clinically raised the suspicion of an ectopic pregnancy. The tube was resected cautiously to ensure the uterus was not exposed to bipolar energy. The ultrasonographic finding of a suspicious ovarian cyst was revealed to be a concealed hematoma located in the Pouch of Douglas secondary to tubal rupture. The tissue was removed in an endo bag without spillage.

Postoperatively, the patient recovered well and was discharged on day 2 postop. The pathology report confirmed the diagnosis of ectopic pregnancy with the presence of chorionic villus and trophoblastic elements in the tube. The intrauterine pregnancy progressed, and the patient delivered a healthy infant weighting 3450gr. via planned caesarean section at 38 weeks.

## Discussion

Heterotopic pregnancy usually is diagnosed at the beginning of pregnancy in women undergoing ART, as the early ultrasonography in these pregnancies may contribute to the diagnosis. Following IVF treatment, the percentage of heterotopic pregnancy may rise to 1-3%, according to different studies, and ultra sonographers should always consider the possibility of HP. The accuracy of diagnosis with ultrasonography varies from 26.2% up to >90% (Li et al., 2013). However, an IUP may actually mask the ectopic component resulting in delayed diagnosis. Moreover, in spontaneous pregnancies, due to the low index of suspicion of a HP, the percentage of severe clinical presentation (tubal rupture-abortion, haemoperitoneum) is higher in this subset of patients (Barrenetxea et al., 2007). Most of the spontaneous HP reported have been treated in the first trimester and only a few cases have been treated in the second trimester. They were all managed by laparotomy, except one reported case performed by laparoscopy (de Oliveira et al., 2021; Gabriel and Taylor, 2021; Okunowo et al., 2016).

It is worth noting that our patient was also a war refugee, which understandably reduced her access to healthcare in early pregnancy. This is a well- known problem during the European refugee crisis and also among marginalised people worldwide (Kohlenberger et al., 2019). Based on the patient’s history, we can assume that a tubal rupture occurred at around eight weeks before her laparoscopy, when the patient perceived the abdominal pain. The hematoma found intraoperatively, was concealed and self-limited, explaining why she remained stable.

Adnexal pathology during pregnancy is not uncommon, especially with the widespread use of ultrasound in early pregnancy. The incidence of adnexal masses in pregnancy is 1% to 6%. Most of these are simple cysts, mainly smaller that 5cm that usually resolve with expectant management. Malignancy is always a possibility, and although less prevalent, it is one of the reasons for elective surgical intervention, with ovarian torsion and cyst rupture being the main indications for emergency surgery. The primary concern in surgery during pregnancy is the potential adverse outcomes such as preterm delivery or miscarriage. Laparoscopy does not seem to induce the prevalence of these complications (Cagino et al., 2021). In our case, the haematoma mimicked a suspicious ovarian cyst, and we proceeded to laparoscopic intervention to explore and treat the pathology. The pregnant uterus, especially in the second trimester, may interfere with the detailed imaging of the adnexa and thus reduce the accuracy of the diagnosis.

Laparoscopy for adnexal pathology during the second trimester of pregnancy may cause several technical difficulties for the surgeon. The reduced space, in combination with difficulty in handling the hyperemic and enlarged uterus (no manipulator is applied) may result in limited access to pathology and the pouch of Douglas in general. Moreover, caution should be taken to avoid possible lacerations to the enlarged uterus with the placement of the Veress needle. All trocars should be placed 5cm cephalad to the normal position to maximise the operating field view. Moreover, an extra lateral trocar could facilitate the surgeon’s access to the adnexa and the pouch of Douglas as demonstrated in our case. Ureteral injury is always a concern when operating by laparoscopy in a narrow field, as in the second trimester of pregnancy. Direct vision of the ureter when applying thermal energy and cutting the retroperitoneal structures when previously dissected are some of the principles the laparoscopic surgeon should follow to avoid ureteral damage. Finally, anaesthesia modifications such as applying deep neuromuscular blockade during the operation may improve the operating field visualisation and reduce post-operative pain and analgesic consumption (Kathopoulis et al., 2022).

## Conclusions

We report a rare case of spontaneous heterotopic pregnancy diagnosed and treated in the second trimester after a ruptured ectopic had caused a haematoma, which on TVS, raised concern for ovarian malignancy. Laparoscopic surgery for adnexal pathology in the second trimester is a demanding and skillful operation. The surgeon should balance the effort to establish maximum operating field visualisation with minimal uterine manipulation and energy application to avoid any potential adverse effects to the intrauterine pregnancy.

## Video scan (read QR)

https://vimeo.com/798328331/990a61b148

**Figure:**